# Refining Structural Analysis of Proteins: Automated Methods to Measure Transition Dipole Strength of Single Residues

**DOI:** 10.1021/acs.jpcb.5c03566

**Published:** 2025-08-06

**Authors:** Dalton R. Boutwell, Amanda L. Cao, Allison S. Walker, Lauren E. Buchanan

**Affiliations:** † Department of Chemistry, 5718 Vanderbilt University, Nashville, Tennessee 37235, United States; ‡ Department of Biological Sciences, Vanderbilt University, Nashville, Tennessee 37235, United States

## Abstract

Transition dipole strength (TDS) analysis enhances two-dimensional infrared (2D IR) spectroscopy by probing protein structural differences that frequency alone cannot resolve. However, its application has been limited to strong signals due to challenges with low signal-to-noise and large backgrounds in the linear optical density. Manual baseline correction can suffer from user error and produce large artifacts that obscure signals of interest. Here, we introduce a new approach incorporating automated baseline correction via the airPLS algorithm to improve the accuracy and precision of TDS calculations across broad spectral windows. Using human islet amyloid polypeptide, we demonstrate TDS analysis of a single ^13^C^18^O-labeled residue, enabling a more precise measure of protein structure at the single-residue level. Further, airPLS-corrected TDS spectra can be calculated throughout amyloid aggregation to resolve potential intermediate structures. This work establishes TDS as a robust tool for investigating the structural dynamics of proteins and other complex macromolecular assemblies.

## Graphical abstract

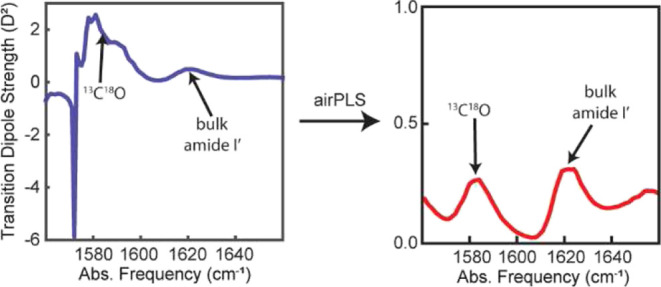

## Introduction

Infrared (IR) spectroscopy can provide a wealth of information on molecular structure and dynamics, including local environment, hydrogen bonding effects, and chemical reaction kinetics.^1^ Commercial Fourier-transform infrared (FT IR) spectrometers provide a fast, nondestructive method to investigate a wide range of systems ranging from organic molecules to inorganic materials and even biological systems.^2^ Two-dimensional infrared (2D IR) spectroscopy is a nonlinear technique that uses three pulses to generate signal. It retains all the advantages of standard FTIR spectroscopy, but can access additional vibrational transitions, reveal detailed structural information through analysis of crosspeaks that form between coupled oscillators, and interrogate dynamics such as vibrational lifetimes, vibrational energy relaxation pathways, and environmental fluctuations.^3–5^ The nonlinear nature of 2D IR results in the relative enhancement of spectral features arising from strong oscillators over weaker ones and, combined with the spreading of peaks over two dimensions, facilitates the analysis of otherwise crowded or unresolved signals within the fingerprint region.^3,6^

2D IR spectroscopy is a particularly powerful tool for studying protein structure, as the backbone amide groups are sensitive reporters of protein secondary structure.^7,8^ Vibrational coupling between backbone amide I′ modes, which primarily comprise carbonyl stretching, results in unique spectral signatures with characteristic frequency shifts: disordered structures absorb at ∼1645–1650 cm^–1^, α-helices absorb across a broad range from 1635 to 1660 cm^–1^, and β-sheets absorb in the 1615–1630 cm^–1^ range with antiparallel β-sheets having an additional weaker absorption at ∼1685 cm^–1^.^1,3,7^ Residue-specific structural information can be acquired with isotope labeling; inserting a ^13^C^18^O into the protein backbone creates a ∼55 cm^–1^ redshift in the amide I′ mode and isolates the spectral signature from that residue from the bulk protein signal.^9,10^ However, the structural information obtained from frequency shifts can be limited when multiple secondary structures appear at the same frequency, which can occur due to overlap of the frequency ranges for disordered and α-helical structures, or when a protein can adopt multiple conformations with sufficiently similar secondary structures that the frequency difference between them is negligible.^11^

Given that frequency alone is not always sufficient to discern changes in protein structure, it is critical to establish complementary metrics for analyzing secondary structure. Recent work has highlighted the sensitivity of transition dipole strength (TDS) to subtle differences in protein structure that do not measurably correlate with frequency shifts.^11–15^ The TDS of a vibrational mode is directly related to its extinction coefficient and, like frequency, is affected by vibrational coupling. When vibrations couple, oscillator strengths redistribute between the delocalized modes. In the case of peptides and proteins, a single, localized amide I′ mode has a TDS of 0.12 *D* ^2^, which is the same value observed for fully disordered structures. This value increases to 0.26–0.55 *D* ^2^ for α-helical structures or even to 0.25–1.25 *D* ^2^ for β-sheets, depending on the size and organization of the β-sheets.^11,12,15^

Critically, while FTIR can be used to measure TDS for uncoupled vibrational modes, it is largely insensitive to changes in TDS that arise from vibrational coupling because the integrated area of the linear absorption spectrum is constant.^3,15^ This is not true for nonlinear spectroscopies, making 2D IR highly sensitive to changes in TDS. Extracting TDS values from 2D IR spectroscopy is more complicated than for FTIR, but Grechko and Zanni developed a method that uses ratios of 2D to 1D IR signals to accurately calculate the absolute TDS of a vibrational mode.^15^ Their approach has been used to differentiate between disordered and α-helical forms of rat amylin,^12^ distinguish between amyloid fibrils formed by human amylin under varying conditions^16^ and even provide label-free detection of two distinct β-sheet polymorphs within samples that appear homogeneous;^13^ in each of these cases, the structural differences between samples were indistinguishable in both FTIR and 2D IR spectra.

These studies clearly demonstrate that TDS measurements are a powerful, nonperturbative approach to discerning protein secondary structure that can be more sensitive than frequency measurements alone. However, there are still practical challenges that must be addressed before TDS calculations are widely adopted by the community. First and foremost, TDS calculations can exhibit a high degree of variability, as demonstrated in a study by Lomont et al. which found that amyloid fibrils formed by the same protein can have TDS values ranging from 0.5 to 1.25 *D* ^2^.^11^ This variability is explained, at least in part, by the fact that amyloid fibrils are highly polymorphic and conformationally sensitive to small changes in aggregation conditions.^17–20^ However, we observe sample-to-sample variations in TDS, albeit over a much smaller range, for small molecules with uncoupled vibrational modes, although the average TDS over multiple samples ultimately approaches the correct value. The second challenge is applying these methods to weak signals, as the need to take ratios of 2D to 1D signals means that low signal-to-noise in the 1D trace can poison the calculation. To date, this has prohibited TDS measurements of ^13^C^18^O labels and other site-specific infrared probes, which have inherently weaker signals than that of the bulk protein amide I′ mode.

In this work, we present a new approach to both improve the precision of TDS calculations and expand their application to single ^13^C^18^O labels. Our approach takes advantage of the adaptive iteratively reweighted penalized least-squares regression (airPLS) algorithm to more reliably correct the background in the linear optical density.^21–23^ The airPLS algorithm has received extensive use in medical imaging, Raman spectroscopy, surface-enhanced Raman scattering, NMR and FTIR spectroscopy, for its ability to unobscure spectral data.^21,24–29^ airPLS eliminates human error in background fitting and can accurately fit to complex backgrounds over a broad spectral range; it also uses internal penalties to automatically avoid fitting to spectral features of interest. For particularly noisy data, a Savitzky–Golay smoothing filter can fit sections of the baseline to individual “moving windows” along the spectra, eliminating more localized perturbations in that spectral range.^30^ Additionally, we examine the efficacy of pump normalization to account for variation in the pump intensity and facilitate TDS analysis of the full amide I′ range.^15^ We demonstrate the increased precision and accuracy from these improvements with TDS analysis of small molecules and establish new calibrants that cover the 1575–1685 cm^–1^ range. Finally, this allows us to leverage TDS analysis to discern local, dynamic structural information by quantifying vibrational delocalization at a single isotopically labeled residue during the aggregation of human islet amyloid polypeptide (hIAPP).^16,31^

## Methods

### Preparation of Fmoc-^13^C^18^O Labeled Valine

Techniques for Fmoc-protection and ^13^C^18^O isotope labeling of free amino acids have been previously established and detailed.^32,33^ Briefly, Fmoc-protection of commercially available ^13^C-labeled valine and ^13^C-labeled phenylalanine was successfully achieved by preparing 1:1:1 ratios of amino acid, Fmoc-Osu, and NaHCO_3_ in a 50:50 water/acetone mixture. The solution was stirred at room temperature for 24 h, before being quenched by 2 M KHSO_4_ to decrease the pH to ∼2.0 and precipitate the product. Vacuum-filtration followed by ice-cold water washes were utilized to remove any salt, with four washes in total and subsequent lyophilization taking place between each wash to remove excess water. ^18^O-isotope labeling was completed via acid-catalyzed ^18^O-isotope exchange of the newly synthesized Fmoc-protected ^13^C-valine and ^13^C-phenylalanine by dissolving 1 g of amino acid with 1 g of ^18^OH_2_ in 8 mL of dioxane plus 4 mL of 4 M HCl in dioxane. The mixture was refluxed under inert conditions at 150 °C for 4 h, then lyophilized to isolate the products. For high labeling efficiency (+90%), the reaction was repeated twice, and the success of the labeling scheme was confirmed with electrospray ionization mass (ESI-MS) spectrometry. Any leftover salts in the product were removed via ether extraction.

### Peptide Synthesis, Cleavage and Purification

Detailed procedures for the Solid Phase Peptide Synthesis (SPPS), cleavage and purification of peptides have been described previously.^32,33^ hIAPP was synthesized via microwave-assisted SPPS with a Liberty Blue peptide synthesizer (CEM, Matthews, NC, USA) using Rink Amide ProTide Resin to produce an amidated C-terminus and pseudoprolines to prevent aggregation of the peptide on the resin, respectively. The peptides were cleaved from resin using 90% trifluoroacetic acid, 5% 1,2-ethanedithiol, 2.5% thioanisole and 2.5% anisole. After stirring the cleavage mixture for 2.5 h at room temperature, the mixture was filtered directly into ice-cold diethyl ether in order to precipitate the hIAPP and remove the resin. Subsequently, hIAPP was centrifuged at 5000 rpm for 5 min and the supernatant was decanted with a glass pipet to remove organic side products. The ether washes were repeated twice with fresh diethyl ether each time. Prior to HPLC purification, hIAPP was dissolved in 20% acetic acid at a concentration of 5.0 mg/mL and then lyophilized for 16 h to remove the solvent. The dried peptide was subsequently dissolved in DMSO at a concentration of 2.5 mg/mL for 48 h to encourage disulfide bond formation between Cys2 and Cys7. Lastly, hIAPP was diluted to 1.25 mg/mL using water, before purification using high-performance liquid chromatography (HPLC, Ultimate 3000, Thermo Fisher, Waltham, MA, USA), using a binary solvent system of ∼100% water/∼0.045% HCl (solvent A) and ∼90% acetonitrile/∼10% water/∼0.045% HCl (solvent B). The gradient was varied from 30% to 45% B over 15 min, while UV absorbance was monitored at 215 and 280 nm; hIAPP eluted at ∼11 min. The eluted peak was characterized using ESI-MS and confirmed to be hIAPP.

### FT IR Spectroscopy and TDS Determination of Calibrants

FT IR spectroscopy was used to experimentally calculate the TDS of small molecules identified for use as calibrants, with the Nicolet IS20 FTIR spectrometer (Thermo Fisher). For each small molecule, a solvent spectrum was collected before the sample spectrum and automatically subtracted by the instrument software to remove the background. Samples of neat DMF, 400 mM 2H5NBA in chloroform, 10 mM NMP in D_2_O, and 120 mM APA in D_2_O were used. The transition dipole moment was determined based on eq 1, where the integral was evaluated through numerical, trapezoidal integration of the peaks associated with the vibrational transitions of interest for each calibrant.^15,34,35^

1 $$D=9.184\times 10^{-3}\int \frac{\epsilon \left(\nu \right)}{\nu }d\nu$$

The beginning and end of a peak for numerical integration was determined by examining the zero-crossings in the first derivative FTIR spectra for each calibrant, and three replicates were taken and averaged for the dipole strengths of each vibration. These calculations were performed using a custom Python script.^36^

### 2D IR Sample Preparation

Samples of DMF, 2H5NBA, NMP, and APA were prepared as for FTIR. l-serine was prepared at concentrations of 5–40 mM in D_2_O. *N*-methylacetamide (NMA) was prepared at 120 mM in D_2_O. ∼1 mM stocks of hIAPP were prepared in dHFIP, with precise concentrations determined via the NanoDrop One Micro-UV–visible Spectrophotometer (Thermo Fisher Scientific, Wilmington, DE, USA). To disaggregate any preformed aggregates, stock solutions were sonicated for 2 h and then allowed to sit at room temperature overnight. Individual samples were prepared for 2D IR aggregation experiments by lyophilizing aliquots of stock solution and dissolving the remaining product in 20 mM deuterated Tris buffer (pD ∼7.6) to a final concentration of 1 mM peptide. For fibril data, hIAPP was allowed to aggregate for a minimum of 2 h to ensure the formation of amyloid fibrils. For kinetic data, 2D IR spectra were collected continuously throughout the aggregation process. All samples were deposited between two CaF_2_ windows with a 50 μm Teflon spacer.

### 2D IR Spectroscopy

Comprehensive details for 2D IR data procurement, treatment, and analysis have been described in prior work.^13,33^ 800 nm pulses (6.7 W, 1 kHz, 60 fs) were generated using a single box ultrafast amplifier (Solstice, SpectraPhysics, Milpitas, CA, USA). Half of the output was used to generate mid-IR light (6150 nm, 25 mW, 1 kHz, <100 fs) via an optical parametric amplifier with difference frequency generation (TOPAS-Prime, SpectraPhysics, Milpitas, CA, USA). The mid-IR beam went into a 2D IR pulse shaper (2DQuick IR, PhaseTech Spectroscopy, Madison, WI, USA) and split into pump and probe beams. The signal was dispersed by a monochromator (Princeton Instruments, NJ, USA) onto a mercury cadmium telluride focal-plane array detector (PhaseTech Spectroscopy, Madison, WI, USA). PhaseTech QuickControl software was used to collect all data, which was subsequently processed using custom MATLAB codes. 400 mM 4NBA in toluene was used to calibrate the spectrometer. All presented 2D IR spectra were averaged for 20 min. To obtain full aggregation kinetics of hIAPP, signal was averaged for 66 s to generate individual 2D IR spectra. There was an approximately 5 min delay between initiating aggregation and obtaining the first 2D IR spectrum, after which spectra were collected continuously for approximately 2.5 h.

### TDS Calculations from 2D IR Spectroscopy

Standard calculation of TDS values from 2D IR spectroscopy has been described in the literature and is provided in eq 2.^12,13,15^

2 $$d\left(\omega \right)=\frac{\frac{\Delta OD_{sample}\left(\omega ,\omega \right)}{OD_{sample}\left(\omega \right)}}{\frac{\Delta OD_{calibrant}\left(\omega_{\max},\omega_{\max}\right)}{OD_{calibrant}\left(\omega_{\max}\right)}}\cdot {\left|\mu \right|}_{calibrant}^{2}\cdot \frac{I_{pump}\left(\omega_{\max}\right)}{I_{pump}\left(\omega \right)}$$

The change in optical density (ΔOD) is obtained directly from the diagonal intensity slice of a 2D IR spectrum. The linear optical density (OD) is calculated from the transmission spectrum of the 2D IR probe pulse.^12^

The ratio of ΔOD/OD is calculated across the spectral region of interest for the sample. The absolute TDS is obtained by scaling this ratio to a known calibrant, first by dividing by the same ΔOD/OD ratio calculated at the frequency of peak maximum­(ω_max_) for the calibrant and then multiplying by the square of the magnitude of the calibrant transition dipole moment (|μ|_calibrant_^2^). The final term, a ratio of pump intensities, is typically neglected when analyzing bulk amide I′ signals; however, we will discuss its importance for analyzing vibrational modes across a broad frequency range.

A spectrum of deuterated 20 mM Tris buffer was collected to subtract the solvent background from the linear OD for peptide samples. NMA was primarily used as the calibrant for peaks in the standard amide I′ spectral range, while 2H5NBA was used as the calibrant for analysis of ^13^C^18^O isotope labeled peaks. Pump spectra were collected by removing the sample and steering the pump beam directly into the monochromator. If using a solvent with significant background absorption in the same spectral region, the pump spectrum should be collected with a solvent sample in place. Data was processed using a custom MATLAB script. To measure changes in TDS during hIAPP aggregation, ∼16.5 min of 2D IR data (15 spectra) were averaged to improve signal-to-noise before extracting the linear OD and ΔOD.

### AirPLS Baseline Correction, Optical Density Smoothing and Pump Intensity Normalization

The Adaptive Iteratively Reweighted Penalized Least Squares (airPLS) regression algorithm^21,22^ was used for precise baseline subtraction in the linear OD, with the smoothness parameter adjusted based on signal strength. The algorithm is an automated procedure that gradually readjusts until it fits a complex baseline for background subtraction. The weights of iteration along the baseline at different points are adapted and adjusted based on the sum square errors (SSE) between the previously fitted baseline and the original signal (s). To manipulate the smoothness of the baseline, a penalty is introduced based on the sum square derivatives of the adjusted baseline, and both of these aspects help the algorithm to automatically adjust the background while avoiding a bias toward fitting the baseline to actual peaks. This allows the entire airPLS algorithm to correct the baseline across the entire spectral window of the linear OD without the need to manually exclude peaks or regions of high noise that would lead to a poor background fit. The baseline is adjusted and reweighted iteratively until the maximum number of iterations are reached, or ideally when the terminative criterion of the procedure is reached, or when the differences between the baseline vector and the original signal vector are less than 0.1% of the original signal vector. In some cases with particularly noisy data or weak signal, a Savitzky–Golay smoothing filter from the signal processing toolbox in MATLAB was used to further smooth and process the data, by discretely convoluting the linear optical density and fitting windowed sections of it to individualized polynomials for subtraction.^30^ The Savitzky–Golay smoothing filter relies on linear least-squares regression and a third order polynomial was used for each window (each consisting of 15 data points). We assessed the effect of normalizing for frequency-dependent differences in pump intensity (the final term in eq 2) on the TDS analysis of vibrational modes absorbing more than 20 cm^–1^ from the ω_max_ of the calibrant molecule. For comparison, TDS calculations were also completed by manually selecting which points to include or exclude (such as those from apparent/expected peak locations) before fitting a second or third order polynomial function to the linear OD baseline. Multiple fits, using both second and third order polynomials and different frequency ranges for the baseline, were attempted in the manual background subtraction of the linear OD for each sample and the best fit was chosen for comparison to airPLS.

## Results & Discussion

We first examined the robustness of the airPLS algorithm on a simple model system with no vibrational coupling: the carboxyl stretching mode of l-serine, which appears at ∼1623 cm^–1^ and has a well-documented TDS value of 0.200 *D* ^2^. We compared standard polynomial background correction with airPLS across a range of concentrations to determine whether airPLS improves the precision of the TDS values obtained, even at concentrations where low signal-to-noise typically prohibits TDS analysis. The differing signal-to-noise ratios for 40 mM and 10 mM l-serine are clear in 2D IR spectra (Figure 1A,B). While the respective diagonal intensity slices have similar lineshapes and backgrounds (Figure S1) despite the reduced signal in the 10 mM sample, the linear OD spectra exhibit (Figure 1C,D) dramatically different backgrounds and noise levels. Even for 40 mM l-serine, a concentration that has been used to calibrate TDS spectra in prior work,^12^ manual background correction using a polynomial fit (Figure 1C, blue) leads to more frequent zero-crossings in the corrected linear OD (Figure S2A, blue). While a background correction with residuals centered around zero would typically be considered ideal, dividing by the corrected linear OD in eq 2 creates large noise artifacts in the calculated TDS spectra (Figure 1E, blue) whenever the baseline is zero. In contrast, background correction with airPLS avoids these zero-crossings and produces a flat baseline with a slight positive offset (Figure S2A, red). The TDS calculation, which normalizes the sample ΔOD/OD ratio by the calibrant ΔOD/OD (eq 2), automatically corrects for this remaining offset and yields a TDS spectrum with both accurate peak values and significantly reduced noise (Figure 1E, red). The advantages of airPLS are even more apparent at 10 mM, where lower signal-to-noise results in noise spikes that distort the carboxyl peak in the TDS spectrum when the background is corrected manually (Figure 1D,F, blue), while airPLS yields a clean TDS spectrum (Figure 1D,F, red). In fact, the 10 mM TDS spectrum from airPLS closely matches its 40 mM counterpart, despite having both a weaker peak and a larger, more sloped background.

**Figure 1:**
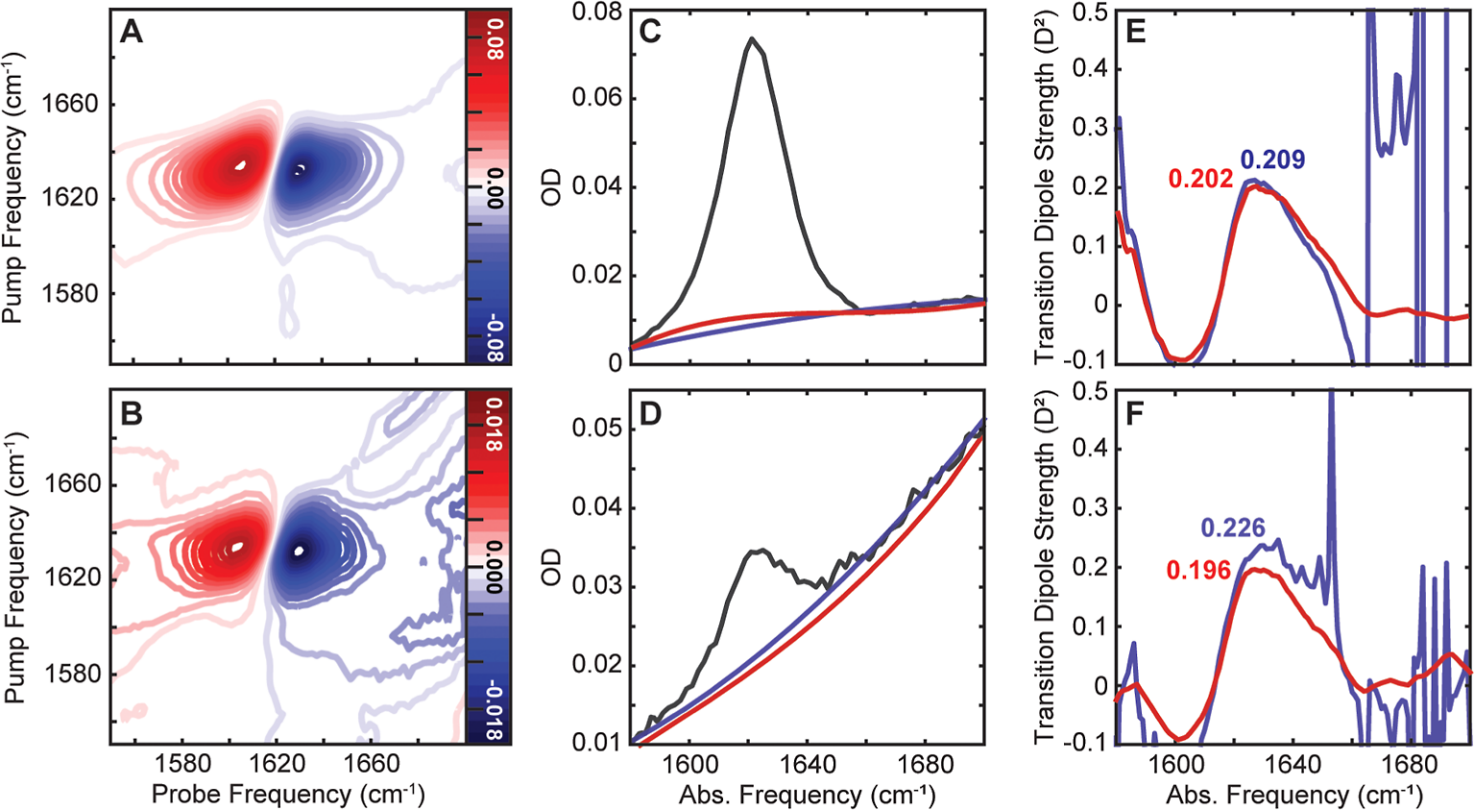
Comparison of manual and airPLS background correction for TDS analysis of l-serine. 2D IR (A,B), linear OD (C,D), and final TDS (E,F) spectra of 40 mM (top row) and 10 mM (bottom row). Manual polynomial fits (blue) and airPLS fits (red) to the baseline are shown on the linear OD spectra. airPLS was applied to the entire linear OD spectrum, while manual fitting applied a 2nd order polynomial to the baseline at 1550–1590 and 1660–1748 cm^–1^. TDS spectra calculated from the airPLS-corrected linear OD spectra (red) exhibit lower noise and higher accuracy than those obtained from the manually corrected linear OD spectra (blue).

We also attempted to calculate the TDS spectrum of 5 mM l-serine. Manual baseline correction results in significant noise across the main TDS peak (Figure S3, blue), precluding accurate TDS analysis. Using airPLS considerably reduces noise in the TDS spectrum, but does not fully eliminate it (Figure S3, red). Finally, we applied a Savitzky–Golay filter in addition to airPLS to remove noise in the linear OD. This eliminated all but one artifact from the TDS spectrum, which is offset from the carboxyl peak (Figure S3, green).

For all concentrations tested, the accuracy of the TDS measurement dramatically improved with airPLS (Table 1). If we take the TDS value for each spectrum at the maximum of the carboxyl peak, manual polynomial fitting yielded TDS values of 0.209–0.226 *D* ^2^ for l-serine concentrations between 10 −40 mM, representing 5 −15% error relative to the established value of 0.200 *D* ^2^. Using airPLS reduced this error to 2–3% for the same samples, or TDS values of 0.195–0.202 *D* ^2^. Across all samples, the standard deviation in TDS decreased by nearly 60%, indicating that airPLS also improved the precision of the TDS calculations. For 5 mM l-serine, the lowest concentration tested, manual baseline correction yielded a TDS value of 0.284 *D* ^2^ (42% error) which improved to 0.256 *D* ^2^ (28% error) with airPLS and to 0.241 *D* ^2^ (21% error) with airPLS plus noise filtering. While the airPLS results show dramatic improvement over manual fitting, even the most accurate TDS value for 5 mM l-serine is an outlier according to Grubbs’ test.^37^ This suggests a lower limit for accurate TDS analysis when the linear OD has a signal-to-noise ratio between 2.45 (5 mM l-serine) and 4.58 (10 mM l-serine).

**Table 1:** Comparison of TDS Values for l-Serine in D_2_O, Using Either Manual Polynomial Fitting or airPLS to Correct the Linear OD Background^a^

	manual		airPLS	
concentration (mM)	TDS (*D* ^2^)	% error	TDS (*D* ^2^)	% error
40	0.209	4.5%	0.202	1%
20	0.222	11%	0.195	2.5%
10	0.226	13%	0.196	2%
5	0.284	42%	0.256 (0.241**)	28% (21%**)
mean*	0.219	9.5%	0.198	1%
standard deviation*	0.007		0.003	

a *5 mM data not included in calculation of mean or standard deviation. **Savitzky–Golay noise filtering used in addition to airPLS.

The l-serine data clearly demonstrates that, relative to manual background correction, airPLS yields more accurate and precise TDS values and permits TDS analysis of weak signals. Next, we extend these studies beyond model small molecules to analyze vibrational delocalization between coupled oscillators in proteins. hIAPP is a 37-residue peptide that can misfold and aggregate into amyloid fibrils associated with type II diabetes. hIAPP monomers within the fibrils adopt a U-shaped structure comprising two β-sheets connected by a disordered loop (Figure S4).^38^ Incorporation of ^13^C^18^O isotope labels at individual residues allow us to probe structural changes at specific positions within the peptide backbone. To determine whether airPLS can enable TDS analysis of the labels, we synthesized hIAPP with a ^13^C^18^O-labeled valine-17 (V17), which participates in the N-terminal β-sheet of the fibrils. First, we start with fully formed fibrils, when the V17 should be strongly coupled and thus have the largest signal. Manual polynomial fitting could not accurately fit the linear OD baseline across the full spectral region (Figure 2B, blue) and cut through the main amide I′ peak at ∼1620 cm^–1^. The resulting TDS spectrum is noisy and lacks a clearly defined V17 feature around 1582 cm^–1^ (Figure 2C, blue), where it is clearly observed in the 2D IR spectra (Figure 2A). Further, the TDS value obtained for the main amide I′ peak are 2–3 times higher than published values for 1 mM hIAPP, which were obtained by fitting only a small spectral window directly around the amide I′ peak.^12,16^ This emphasizes the need for a better approach to fit the linear OD baseline across a broad spectral window so that both isotope-labeled and bulk amide I′ signals can be analyzed simultaneously. The signal-to-noise ratio for the V17 isotope peak is 5.19, which suggests that accurate TDS values can be obtained with the addition of airPLS.

**Figure 2:**
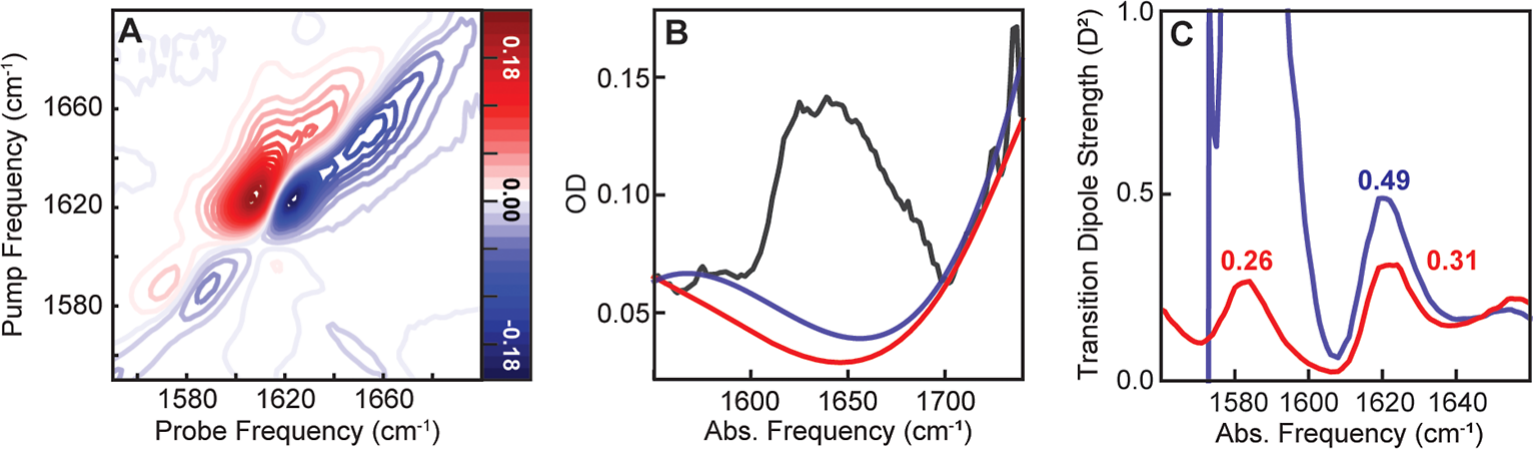
Comparison of manual and airPLS background correction for TDS analysis of 1 mM V17-hIAPP. (A) 2D IR spectrum showing the V17 peak at 1582 cm^–1^. (B) Linear OD with manual polynomial fitting (blue) and airPLS fitting (red) of the baseline. airPLS was applied to the entire linear OD spectrum, while the manual fitting applied a 3rd order polynomial to the baseline at 1550–1570 and 1705–1748 cm^–1^. (C) Corresponding TDS spectra show that TDS analysis of the V17 label is only possible using airPLS.

airPLS accurately fits the linear OD baseline across the entire spectral region from 1500 to 1750 cm^–1^ (Figure 2B, red). The TDS value for the 1620 cm^–1^ peak is now consistent with the literature and, most importantly, a clean V17 isotope peak is visible. The V17 TDS value of 0.26 *D* ^2^ is reasonable as it falls at the lower end of the range of published TDS values for bulk β-sheets and is slightly lower than the value of 0.31 *D* ^2^ obtained for the bulk amide I′ mode.^11,16^ An example of TDS analysis for a noisier V17-hIAPP spectrum is shown in Figure S5 and yields comparable values. This represents the first time that the TDS has been measured for a single residue within a protein.

However, to ensure the accuracy of TDS analysis for vibrational modes across a broader range of frequencies, we must consider whether there is a frequency dependence to the TDS calculation. The broadband pulses used to generate 2D IR spectra do not have flat intensity profiles. When calculating TDS spectra according to eq 2, the full spectrum for the sample is scaled by the TDS value for the calibrant molecule at only a single frequency, the peak maximum. Thus, while the intensity profile of the probe pulse is accounted for by taking the ratio of ΔOD/OD, the intensity profile of the pump pulses is not. We can normalize for the pump spectrum by multiplying the standard TDS equation by $\frac{I_{pump}\left(\omega_{\max}\right)}{I_{pump}\left(\omega \right)}$ ,^15^ although this is typically neglected for amide I′ TDS analysis as established calibrant molecules are sufficiently close in frequency (<20 cm^–1^) to the modes of interest. We identified a set of small molecules with vibrational modes spanning 1585–1685 cm^–1^ (Figure S6), the spectral window in which all protein amide I′ and ^13^C^18^O-labeled amide I′ modes appear, and used FTIR to measure their TDS (Table S1). To calibrate the TDS spectra in Figures 2 and S5, we used the aromatic stretch of 2-hydroxy-5-nitrobenzaldehyde (2H5NBA) at 1585 cm^–1^. This aligns well with the V17 mode but is 38 cm^–1^ lower than the unlabeled amide I′ mode at 1623 cm^–1^.

Here, we examine whether individual calibrants must be closely matched to each mode analyzed or pump normalization is sufficient to ensure accurate TDS values over a broad frequency range. To do so, we compare TDS spectra of V17-hIAPP calculated from the same 2D IR data; the only difference between each spectrum is the calibration method. First, we compare spectra calibrated to either the 1585 cm^–1^ mode of 2H5NBA (Figure 3A) or the 1623 cm^–1^ mode of NMA (Figure 3B). The calculated TDS values differ by 2% for the bulk amide I′ peak and 5% for the V17 isotope peak. While this difference is not significantly larger than the error observed for the airPLS-corrected TDS analysis of l-serine (Table 1), our pump spectrum is relatively flat between these two spectral features (Figure S7). However, we anticipate that calibrant choice would skew the TDS spectrum more significantly as you move further away from the center of the pump spectrum. Next, we compare a TDS spectrum also calibrated with NMA but with the additional factor for pump normalization (Figure 3C). As expected, the TDS value for the bulk amide I′ agrees between the two spectra calibrated between NMA, but pump correction restores the TDS value for the V17 isotope peak to the value obtained when using 2H5NBA. Additionally, the spectral region above 1630 cm^–1^ is noticeably lower after pump normalization is applied; most notably, the TDS at 1645 cm^–1^ decreases from 0.20 *D* ^2^ to 0.13 *D* ^2^. Both disordered and α-helical structures can absorb at this frequency, but disordered structures should have a TDS of 0.12 *D* ^2^ while α-helices will be higher. As hIAPP fibrils contain disordered regions but no α-helices, we conclude that pump normalization is critical to accurately producing full TDS spectra.

**Figure 3:**
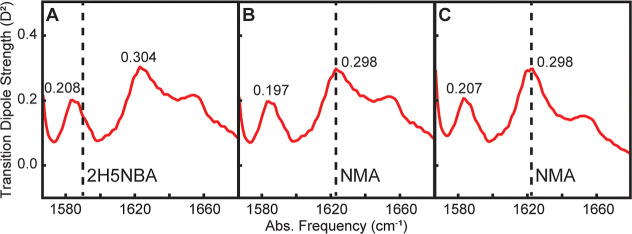
TDS spectra of 1 mM V17 hIAPP fibrils calibrated using (A) 2H5NBA, (B) NMA, and (C) NMA with pump normalization. The frequency of the ω_max_ for the calibrant molecules is indicated with a dashed line.

Uncovering the TDS of amide I′ modes from individual isotope-labeled residues provides crucial insight into local protein structure. However, protein structure can change in response to stimuli or as a result of self-assembly. To improve our understanding of these dynamic processes, TDS analysis must be capable of distinguishing these time-dependent changes to structure. In prior work, we have shown that TDS spectra of the bulk amide I′ mode can detect differences in β-sheet polymorphs that cannot be resolved using frequency alone.^13^ Here, we demonstrate that airPLS-corrected TDS analysis can track both the aggregation of hIAPP and the incorporation of V17 into the fibril structure. Traditionally, the intensity of the β-sheet amide I′ mode at 1623 cm^–1^ is used to track fibril growth (Figure 4, black), similar to the kinetic traces provided by thioflavin-T fluorescence experiments.^39^ The kinetic trace displays a sigmoidal shape with an initial lag phase, a period of rapid growth, and a final equilibration phase. The intensity during the equilibration phase often continues to increase slowly, possibly from continued lengthening or remodeling of the fibrils.^39–41^ We observe similar trends in the TDS of both the bulk amide I′ mode (Figure 4, red) and the V17 isotope mode (Figure 4, blue). Notably, V17 has an initial TDS of 0.13 *D* ^2^ during the lag phase, indicating it is primarily disordered, while the TDS of the bulk amide I′ mode is higher at 0.26 *D* ^2^, indicating that at least part of hIAPP has already adopted a β-sheet structure. This agrees with an aggregation mechanism reported in the literature, which proposes that hIAPP can form oligomer β-sheets between residues 23–27 during the lag phase while the rest of the peptide chain, including V17, remains disordered.^39,41,42^

**Figure 4:**
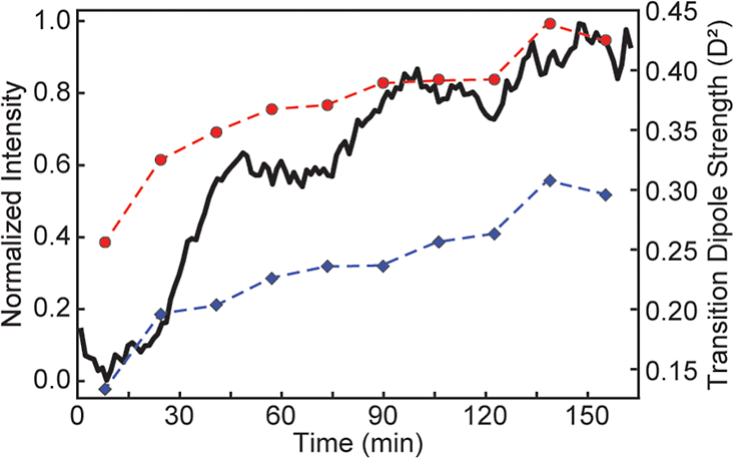
Aggregation kinetics for 1 mM V17 hIAPP, based on intensity of β-sheet amide I′ signal at 1623 cm^–1^ (black), the TDS of the 1623 cm^–1^ mode (red circles), and the TDS of the V17 isotope-labeled amide I′ mode at 1582 cm^–1^ (blue diamonds). Each TDS data point is calculated from the average of 15 2D IR spectra and positioned in the middle of each corresponding ∼16.5 min window.

## Conclusions

In this work, we demonstrate that integrating airPLS into TDS calculations shifts TDS analysis from a sensitive, and yet bulk, technique capable of revealing subtle differences in global protein structure to a precise tool for examining the localized structural changes. By eliminating user error from manual baseline fitting and suppressing artifacts that arise due to low signal-to-noise, airPLS both improves the accuracy and precision of TDS calculations and enables, for the first time, their application to individual ^13^C^18^O-labeled residues. Applying a Savitzky–Golay smoothing filter can further reduce artifacts in noisy spectra, but care must be taken to avoid oversmoothing the TDS spectra and potentially losing fine structure indicative of heterogeneity.^13^ Including a pump normalization term in the calculation ensures that TDS spectra are well-calibrated across the full amide I′ range without the need to employ multiple calibration standards.

Our group and others have shown previously that the TDS of the amide I′ mode can be more sensitive to differences in protein structure than frequency alone.^11–16^ We expect that same to be true for individual isotope-labeled residues. We have demonstrated, for the first time, TDS analysis of single ^13^C^18^O labels. Further, we have shown that this analysis does not require any modification of experimental conditions—it can be performed using the same sample concentrations and data collection times used in typical 2D IR studies of proteins. The ability to track the TDS of individual residues in real-time as proteins sample different conformations provides researchers with unprecedented access to understanding the ensemble of short-lived and polymorphic oligomeric species implicated in amyloid disease that are largely inaccessible to traditional techniques such as cryo-EM. Ultimately, we anticipate that more robust and accurate TDS analysis will serve as a powerful tool to unravel the structural heterogeneities of a wide range of biological and synthetic macromolecules.

## Supplementary Material



## Notes

Includes detailed materials and methods, a table of small molecule infrared frequencies and TDS values, and additional spectra (PDF)

## References

[1] Barth A. (2007). Infrared Spectroscopy
of Proteins. Biochim. Biophys. Acta, Bioenerg..

[2] Griffiths, P. R. ; de Haseth, J. A. Fourier Transform Infrared Spectrometry; Wiley, 2006. https://onlinelibrary.wiley.com/doi/epdf/10.1002/047010631X (accessed 2025–05–07)

[3] Hamm, P. ; Zanni, M. Concepts and Methods of 2D Infrared Spectroscopy, 1st ed.; Cambridge University Press, 2011.

[4] Jansen T. L. C., Knoester J. (2009). Waiting Time Dynamics in Two-Dimensional
Infrared Spectroscopy. Acc. Chem. Res..

[5] Woutersen S., Mu Y., Stock G., Hamm P. (2001). Hydrogen-Bond Lifetime Measured by
Time-Resolved 2D-IR Spectroscopy: N-Methylacetamide in Methanol. Chem. Phys..

[6] Hunt N. T. (2024). Using 2D-IR
Spectroscopy to Measure the Structure, Dynamics, and Intermolecular
Interactions of Proteins in H _2_ O. Acc. Chem. Res..

[7] Krimm S., Bandekar J. (1986). Vibrational Spectroscopy and Conformation
of Peptides,
Polypeptides, and Proteins. Adv. Protein Chem..

[8] Ganim Z., Chung H. S., Smith A. W., DeFlores L. P., Jones K. C., Tokmakoff A. (2008). Amide I Two-Dimensional
Infrared Spectroscopy of Proteins. Acc. Chem.
Res..

[9] Ma J., Pazos I. M., Zhang W., Culik R. M., Gai F. (2015). Site-Specific
Infrared Probes of Proteins. Annu. Rev. Phys.
Chem..

[10] Torres J., Kukol A., Goodman J. M., Arkin I. T. (2001). Site-Specific Examination
of Secondary Structure and Orientation Determination in Membrane Proteins:
The Peptidic13C=18O Group as a Novel Infrared Probe. Biopolymers.

[11] Lomont J. P., Ostrander J. S., Ho J. J., Petti M. K., Zanni M. T. (2017). Not All
β-Sheets Are the Same: Amyloid Infrared Spectra, Transition
Dipole Strengths, and Couplings Investigated by 2D IR Spectroscopy. J. Phys. Chem. B.

[12] Dunkelberger E. B., Grechko M., Zanni M. T. (2015). Transition Dipoles
from 1D and 2D
Infrared Spectroscopy Help Reveal the Secondary Structures of Proteins:
Application to Amyloids. J. Phys. Chem. B.

[13] Weeks W. B., Buchanan L. E. (2022). Label-Free Detection
of β-Sheet Polymorphism. J. Phys. Chem.
Lett..

[14] Antevska A., Hess K. A., Long C. C., Walker E. J., Jang J. H., DeSoto R. J., Lazar Cantrell K. L., Buchanan L. E., Do T. D. (2024). Deciphering
the Molecular Dance: Exploring the Dynamic Interplay Between Mouse
Insulin B9–23 Peptides and Their Variants. Biochemistry.

[15] Grechko M., Zanni M. T. (2012). Quantification of Transition Dipole Strengths Using
1D and 2D Spectroscopy for the Identification of Molecular Structures
via Exciton Delocalization: Application to α-Helices. J. Chem. Phys..

[16] Hess K. A., Spear N. J., Vogelsang S. A., Macdonald J. E., Buchanan L. E. (2023). Determining the Impact of Gold Nanoparticles
on Amyloid
Aggregation with 2D IR Spectroscopy. J. Chem.
Phys..

[17] Tycko R. (2015). Amyloid Polymorphism:
Structural Basis and Neurobiological Relevance. Neuron.

[18] Caughey B., Raymond G. J., Bessen R. A. (1998). Strain-Dependent
Differences in β-Sheet
Conformations of Abnormal Prion Protein. J.
Biol. Chem..

[19] Heise H., Hoyer W., Becker S., Andronesi O. C., Riedel D., Baldus M. (2005). Molecular-Level Secondary Structure,
Polymorphism, and Dynamics of Full-Length α-Synuclein Fibrils
Studied by Solid-State NMR. Proc. Natl. Acad.
Sci. U.S.A..

[20] Paravastu A. K., Qahwash I., Leapman R. D., Meredith S. C., Tycko R. (2009). Seeded Growth
of β-Amyloid Fibrils from Alzheimer’s Brain-Derived Fibrils
Produces a Distinct Fibril Structure. Proc.
Natl. Acad. Sci. U.S.A..

[21] Zhang Z. M., Chen S., Liang Y. Z. (2010). Baseline Correction Using Adaptive
Iteratively Reweighted Penalized Least Squares. Analyst.

[22] Eilers, P. H. C. ; Boelens, H. F. M. Baseline Correction with Asymmetric Least Squares Smoothing; Leiden University Medical Centre: The Netherlands, 2005. https://prod-dcd-datasets-public-files-eu-west-1.s3.eu-west-1.amazonaws.com/dd7c1919-302c-4ba0-8f88-8aa61e86bb9d (accessed 2025–05–07)

[23] Eilers P. H. C. A. (2003). Perfect
Smoother. Anal. Chem..

[24] Wang Y., Kang S., Doerksen J. D., Glaser A. K., Liu J. T. C. (2016). Surgical
Guidance via Multiplexed Molecular Imaging of Fresh Tissues Labeled
with SERS-Coded Nanoparticles. IEEE J. Sel.
Top. Quantum Electron..

[25] Zheng X., Wu G., Wang J., Yin L., Lv X. (2022). Rapid Detection of
Hysteromyoma and Cervical Cancer Based on Serum Surface-Enhanced Raman
Spectroscopy and a Support Vector Machine. Biomed.
Opt. Express.

[26] Zhang J., Yang Y., Feng X., Xu H., Chen J., He Y. (2020). Identification of Bacterial Blight
Resistant Rice Seeds Using Terahertz
Imaging and Hyperspectral Imaging Combined With Convolutional Neural
Network. Front. Plant Sci..

[27] Hamade K., Fliniaux O., Fontaine J. X., Molinié R., Otogo Nnang E., Bassard S., Guénin S., Gutierrez L., Lainé E., Hano C. (2021). Nmr and
Lc-Ms-Based Metabolomics to Study Osmotic Stress in Lignan-Deficient
Flax. Molecules.

[28] Crusio, W. E. ; Radeke, H. H. Advances in Experimental Medicine and Biology; Springe, 2025; Vol. 1280. http://www.springer.com/series/5584. Date accessed: 1/21/2025

[29] Barretto C.
T., Nascimento M. H. C., Brun B. F., da Silva T. B., Dias P. A. C., Silva C. A. B., Singh M. N., Martin F. L., Filgueiras P. R., Romão W. (2024). Infrared Spectroscopy as a New Approach for
Early Fabry Disease Screening: A Pilot Study. Orphanet J. Rare Dis..

[30] Savitzky A., E M.
J. (1964). Smoothing and Differentiation
of Data by Simplified Least Squares
Procedures. Anal. Chem..

[31] Hess K. A., Rohler C. K., Boutwell D. R., Snyder J. M., Buchanan L. E. (2024). Suppressing
Sidechain Modes and Improving Structural Resolution for 2D IR Spectroscopy
via Vibrational Lifetimes. J. Chem. Phys..

[32] Marek P., Woys A. M., Sutton K., Zanni M. T., Raleigh D. P. (2010). Efficient
Microwave-Assisted Synthesis of Human Islet Amyloid Polypeptide Designed
to Facilitate the Specific Incorporation of Labeled Amino Acids. Org. Lett..

[33] Middleton C. T., Woys A. M., Mukherjee S. S., Zanni M. T. (2010). Residue-Specific
Structural Kinetics of Proteins through the Union of Isotope Labeling,
Mid-IR Pulse Shaping, and Coherent 2D IR Spectroscopy. Methods.

[34] Kubelka J., Keiderling T. A. (2001). Ab Initio Calculation of Amide Carbonyl Stretch Vibrational
Frequencies in Solution with Modified Basis Sets. 1. JV-Methyl Acetamide. J. Phys. Chem. A.

[35] Ackels L., Stawski P., Amunson K. E., Kubelka J. (2009). On the Temperature
Dependence of Amide I Intensities of Peptides in Solution. Vib. Spectrosc..

[36] Van Rossum, G. ; Drake, F. L. Python 3 Reference Manual, 1st ed.; CreateSpace: Scotts Valley, CA, 2009; Vol. 1.

[37] Grubbs F.
E. (1950). Sample
Criteria for Testing Outlying Observations. Ann. Math. Stat..

[38] Luca S., Yau W. M., Leapman R., Tycko R. (2007). Peptide Conformation
and Supramolecular Organization in Amylin Fibrils: Constraints from
Solid-State NMR. Biochemistry.

[39] Buchanan L. E., Dunkelberger E. B., Tran H. Q., Cheng P. N., Chiu C. C., Cao P., Raleigh D. P., De Pablo J. J., Nowick J. S. (2013). Mechanism
of IAPP Amyloid Fibril Formation Involves an Intermediate with a Transient
β-Sheet. Proc. Natl. Acad. Sci. U.S.A..

[40] Shim S.-H., Gupta R., Ling Y. L., Strasfeld D. B., Raleigh D. P., Zanni M. T. (2009). Two-Dimensional
IR Spectroscopy and
Isotope Labeling Defines the Pathway of Amyloid Formation with Residue-Specific
Resolution. Proc. Natl. Acad. Sci. U.S.A..

[41] Buchanan L. E., Maj M., Dunkelberger E. B., Cheng P. N., Nowick J. S., Zanni M. T. (2018). Structural Polymorphs
Suggest Competing Pathways for
the Formation of Amyloid Fibrils That Diverge from a Common Intermediate
Species. Biochemistry.

[42] Serrano A. L., Lomont J. P., Tu L. H., Raleigh D. P., Zanni M. T. (2017). A Free
Energy Barrier Caused by the Refolding of an Oligomeric Intermediate
Controls the Lag Time of Amyloid Formation by HIAPP. J. Am. Chem. Soc..

