# Supplementary material for: Refining Structural Analysis of Proteins: Automated Methods to Measure Transition Dipole Strength of Single Residues
Source: J Phys Chem B. 2025 Aug 6;129(33):8360–7. doi: 10.1021/acs.jpcb.5c03566 (PMC12376093; doi:10.1021/acs.jpcb.5c03566)
Supplement: Supplementary file 1 [file jp5c03566_si_001.pdf]

SUPPLEMENTARY INFORMATION

Refining Structural Analysis of Proteins: Automated Methods to Measure Transition Dipole Strength of Single Residues

Dalton R. Boutwell[1], Amanda L. Cao[1], Allison S. Walker[1,2], Lauren E. Buchanan[1,]*

[1]Department of Chemistry, Vanderbilt University, Nashville, TN, United States 37235

[2]Department of Biological Sciences, Vanderbilt University, Nashville, TN, United States 37235

*Corresponding Author: lauren.e.buchanan@vanderbilt.edu

SUPPORTING DATA

Table S1. Experimental absorption frequencies and transition dipole strengths for small molecules with modes spanning the amide I' spectral region and known ranges for the amide I' mode in peptides and proteins.[1,2] Citations for literature values given in brackets.

**Molecule**	**Mode Assignment**	**Frequency (cm^{-1})**	**Average TDS (D^2)**	
			**This Study**	**Literature**
**Small molecules (TDS obtained from FTIR)**				
NMA	Amide I'	1624	0.120	0.12 [3,4]
DMF	Amide I'	1685	0.137	0.138 [5]
L-Serine	C=O stretch	1623	0.200	0.200 [5]
NMP	Amide I'	1645	0.230	-
APA ω_1	Amide I'	1607	0.173	-
APA ω_2	Amide I'	1652	0.117	-
2H5NBA ω_1	Ar. Stretch	1585	0.0374	-
2H5NBA ω_2	Ar. Stretch	1630	0.0686	-
2H5NBA ω_3	C=O Stretch	1670	0.174	-
**Peptide amide I' modes (TDS calculated from 2D IR)**				
Disordered		1645-1650	-	0.12
α-helix		1635-1660	-	Variable (0.26-0.55) [5,6]
β-sheets	perpendicular to strands (parallel and antiparallel β-sheets)	1615-1630	Variable (0.3-0.49 for hIAPP)	Variable (0.25-1.25) [7–10]
	parallel to strands (antiparallel β-sheets only)	~1685	-	-
$^{13}C=^{18}O$ label		1570-1585	Variable (0.15-0.31 for V17-hIAPP)	-

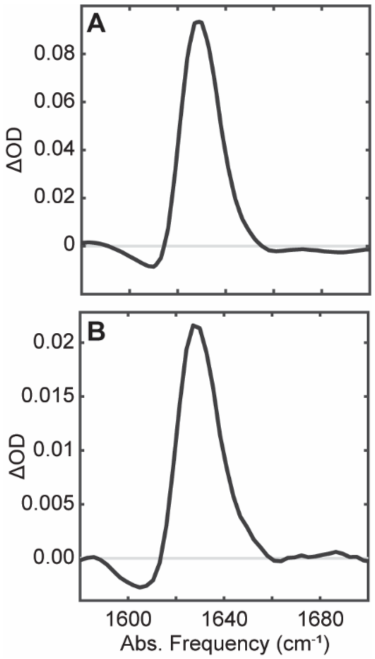

Figure S1. Diagonal intensity slices from 2D IR spectra of 40 mM (A) and 10 mM (B) L-serine.

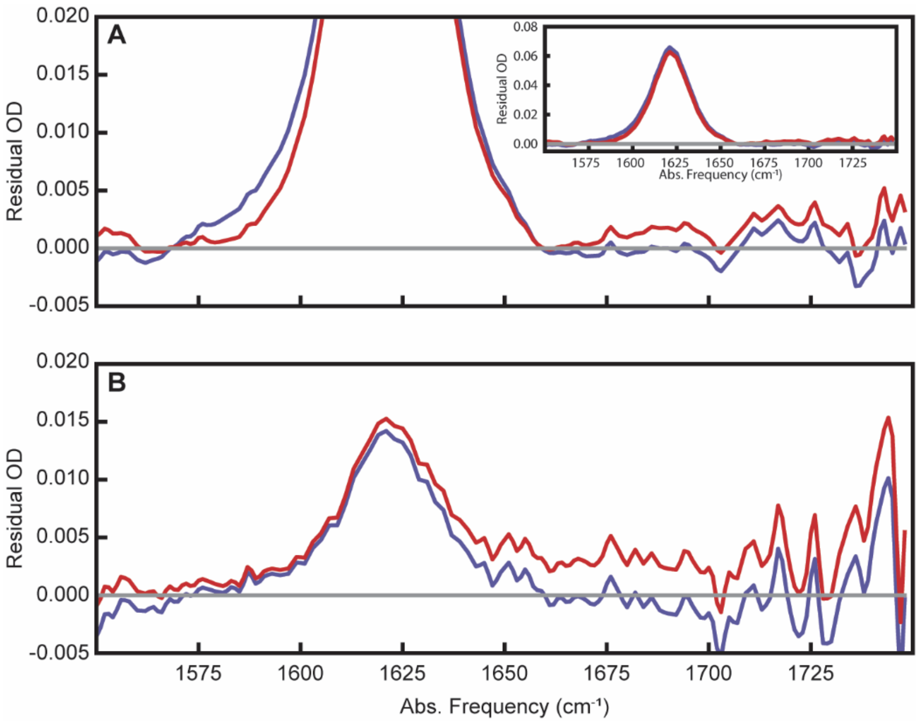

Figure S2. Residual/corrected optical densities for the manual 2nd order polynomial baseline (blue) and airPLS baseline (red) for the 40 mM (A) and 10 mM L-Serine (B) samples shown in **Figure 1**.

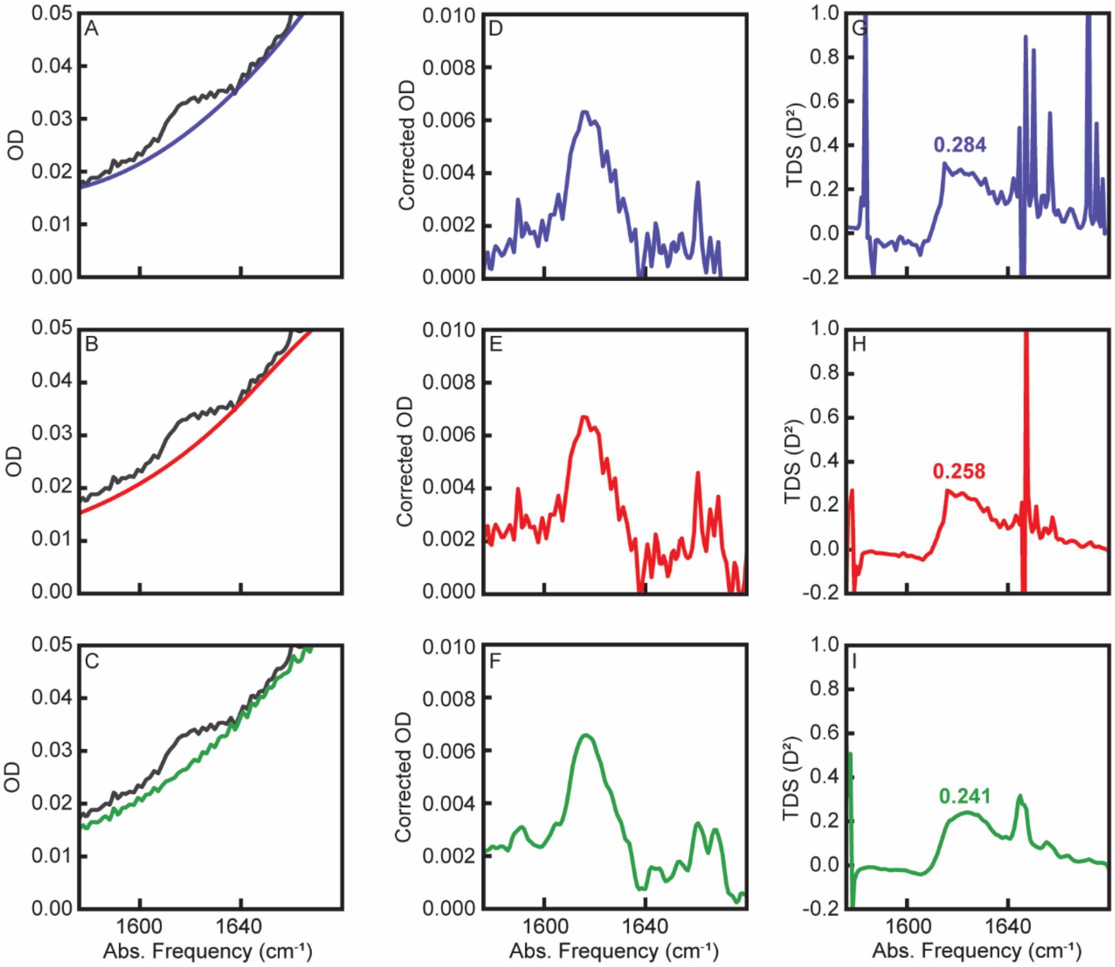

Figure S3. Linear OD (A-C), background-corrected linear OD (D-F), and TDS (G-I) spectra of 5 mM L-Serine using manual polynomial fitting (blue) versus airPLS (red) versus airPLS with Savitsky-Golay noise filtering (green) for background correction.

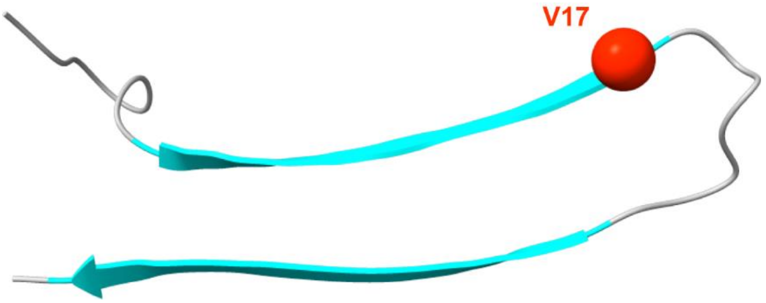

Figure S4. Structure of an hIAPP monomer within the amyloid fibrils, derived from ssNMR.[11] β-sheets are highlighted with cyan arrows and V17 is marked with a red sphere.

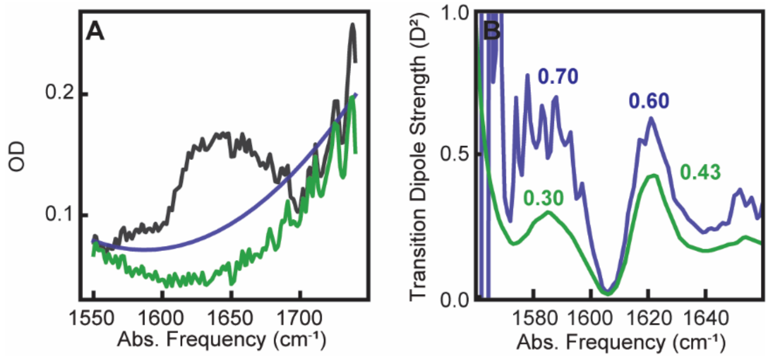

Figure S5. TDS analysis of a V17-hIAPP sample with lower signal to noise. (A) The linear OD spectrum exhibits a signal-to-noise ratio of 3.68 for the V17 peak. Manual polynomial fit (blue) and airPLS fit with Savitsky-Galoy noise filtering (green) of the baseline are shown. (B) Corresponding TDS spectra show that airPLS with noise filtering can produce a TDS spectrum comparable to that obtained for higher signal-to-noise samples.

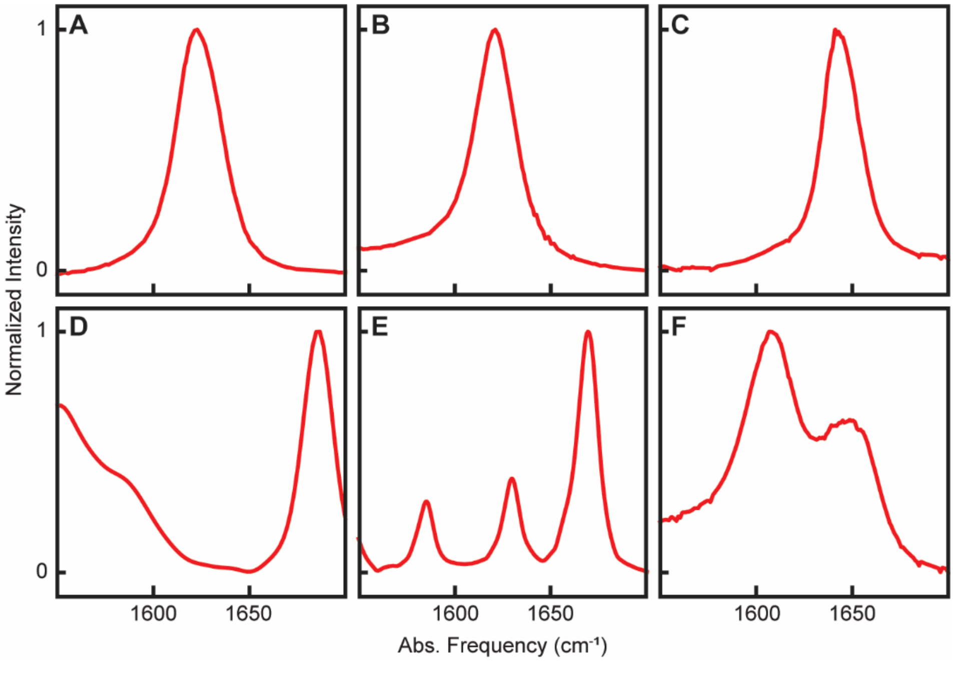

Figure S6. FTIR spectra of potential calibrant molecules: (A) NMA, (B) L-serine, (C) NMP, (D) DMF, (E) 2H5NBA, (F) APA.

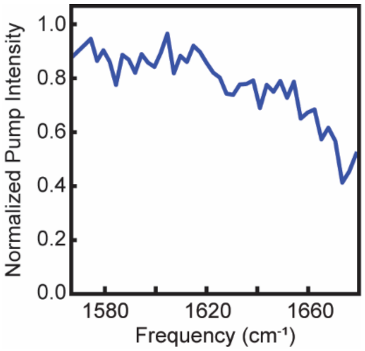

Figure S7. Experimental pump spectrum used to correct the TDS spectra in Fig. 3 according to Eq. 2.

SUPPLEMENTARY REFERENCES

(1) Hamm, P.; Zanni, M. *Concepts and Methods of 2D Infrared Spectroscopy*, 1st ed.; Cambridge University Press, 2011.

(2) Barth, A. Infrared Spectroscopy of Proteins. *Biochimica et Biophysica Acta (BBA) - Bioenergetics* **2007**, *1767* (9), 1073–1101. https://doi.org/10.1016/j.bbabio.2007.06.004.

(3) Ackels, L.; Stawski, P.; Amunson, K. E.; Kubelka, J. On the Temperature Dependence of Amide I Intensities of Peptides in Solution. *Vib Spectrosc* **2009**, *50* (1), 2–9. https://doi.org/10.1016/j.vibspec.2008.07.004.

(4) Kubelka, J.; Keiderling, T. A. Ab Initio Calculation of Amide Carbonyl Stretch Vibrational Frequencies in Solution with Modified Basis Sets. 1. JV-Methyl Acetamide. *Journal of Physical Chemistry A* **2001**, *105* (48), 10922–10928. https://doi.org/10.1021/jp013203y.

(5) Grechko, M.; Zanni, M. T. Quantification of Transition Dipole Strengths Using 1D and 2D Spectroscopy for the Identification of Molecular Structures via Exciton Delocalization: Application to α-Helices. *J Chem Phys* **2012**, *137* (18), 184202. https://doi.org/10.1063/1.4764861.

(6) Dunkelberger, E. B.; Grechko, M.; Zanni, M. T. Transition Dipoles from 1D and 2D Infrared Spectroscopy Help Reveal the Secondary Structures of Proteins: Application to Amyloids. *Journal of Physical Chemistry B* **2015**, *119* (44), 14065–14075. https://doi.org/10.1021/acs.jpcb.5b07706.

(7) Lomont, J. P.; Ostrander, J. S.; Ho, J. J.; Petti, M. K.; Zanni, M. T. Not All β-Sheets Are the Same: Amyloid Infrared Spectra, Transition Dipole Strengths, and Couplings Investigated by 2D IR Spectroscopy. *Journal of Physical Chemistry B* **2017**, *121* (38), 8935–8945. https://doi.org/10.1021/acs.jpcb.7b06826.

(8) Hess, K. A.; Spear, N. J.; Vogelsang, S. A.; Macdonald, J. E.; Buchanan, L. E. Determining the Impact of Gold Nanoparticles on Amyloid Aggregation with 2D IR

Spectroscopy. *Journal of Chemical Physics* **2023**, *158* (9). https://doi.org/10.1063/5.0136376.

(9) Antevska, A.; Hess, K. A.; Long, C. C.; Walker, E. J.; Jang, J. H.; DeSoto, R. J.; Lazar Cantrell, K. L.; Buchanan, L. E.; Do, T. D. Deciphering the Molecular Dance: Exploring the Dynamic Interplay Between Mouse Insulin B9–23 Peptides and Their Variants. *Biochemistry* **2024**, *63* (18), 2245–2256. https://doi.org/10.1021/acs.biochem.4c00217.

(10) Weeks, W. B.; Buchanan, L. E. Label-Free Detection of β-Sheet Polymorphism. *Journal of Physical Chemistry Letters* **2022**, *13* (40), 9534–9538. https://doi.org/10.1021/acs.jpclett.2c02292.

(11) Luca, S.; Yau, W. M.; Leapman, R.; Tycko, R. Peptide Conformation and Supramolecular Organization in Amylin Fibrils: Constraints from Solid-State NMR. *Biochemistry* **2007**, *46* (47), 13505–13522. https://doi.org/10.1021/bi701427q.
